# Fentanyl-induced transformations in composition of lipid droplets in central nervous system cells revealed by ramanomics

**DOI:** 10.1016/j.jlr.2025.100827

**Published:** 2025-05-19

**Authors:** Rahul K. Das, Andrey N. Kuzmin, Artem Pliss, Supriya D. Mahajan, Shobha Shukla, Paras N. Prasad

**Affiliations:** 1 Institute for Lasers, Photonics and Biophotonics and Department of Chemistry, University at Buffalo, The State University of New York, Buffalo, NY, USA; 2 School of Pharmacy, D’Youville University, Buffalo, NY, USA; 3 Department of Medicine, Division of Allergy, Immunology, and Rheumatology, State University of New York at Buffalo, Clinical Translational Research Center, Buffalo, NY, USA; 4 Nanostructures Engineering and Modeling Laboratory, Department of Metallurgical Engineering and Materials Science, Indian Institute of Technology Bombay, Mumbai, MH, India

**Keywords:** cholesterol, kinetics, lipids, lipid/chemistry, phospholipids

## Abstract

Quantitative characterization of the transformations of subcellular molecular environment in response to fentanyl exposure in human microglia and astrocytes is warranted to provide insight into the regulation of neuroinflammatory responses and neural survival in the scenario of opiate drug addiction. Cytoplasmic lipid droplets (LD) act as depot for exogeneous hydrophobic molecules, such as fentanyl, which can lead to increased drug accumulation and alteration of their metabolism. In the present work, we have used an emerging Ramanomics technique that combines quantitative microlipid droplets -Raman spectrometry with biomolecular component analysis to unravel fentanyl induced changes in concentrations of phospholipids, sterols, glycogen, sphingomyelin, phosphocholine as well as RNA and proteins, in the LDs of microglia and astrocytes. The clinical relevance of these findings includes the potential to advance understanding of fentanyl's impact on the central nervous system at a molecular level. The observed alterations in lipid droplet composition, including changes in phospholipids, cholesterol esters, and glycogen accumulation, suggest that fentanyl overdose disrupts cellular homeostasis in microglia and astrocytes. This disruption could contribute to neuroinflammatory responses and impaired neural function, which are critical factors in opioid addiction and withdrawal. By utilizing Ramanomics as a noninvasive, real-time analytical tool, we can better assess fentanyl-induced cellular changes, paving the way for improved diagnostic assays and therapeutic strategies for opioid addiction and overdose treatment.

## Graphical abstract

**Figure:**
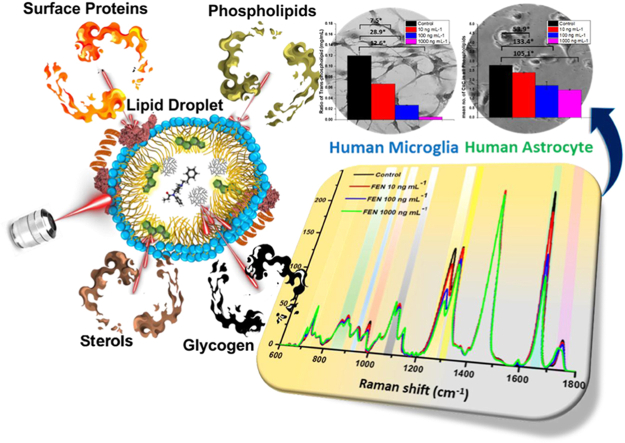

Opioids are powerful painkillers, which target opioid receptors in brain neurons and have been used in medicine since the dawn of civilization. With the advent of modern synthetic chemistry, the chemical structure of opioids has been modified to alter both pharmacokinetics and pharmacodynamics of these drugs. Among the most consequential alterations to the structure of opioids are those which influence their polarity and solubility. A notable example of such modifications is the synthesis of diacetylmorphine, known under Bayer’s trademark as heroine. In this case, the presence of two acetyl groups significantly reduces the polarity of morphine, enhancing intestinal absorption for analgesic applications, but also facilitating crossing through BBB in brain, when heroin is administered by injection into systemic circulation, which makes this drug highly dangerous and addictive. Similar to heroine, fentanyl, another highly potent opioid, exploits high hydrophobicity of its structure, making it soluble in lipids and facilitating its crossing the BBB, which produces the maximum analgesic effect within 5 min following the drug administration. Furthermore, the structure of this drug was modified to enhance interactions with opioid receptors. Under the category of analgesics and anesthetics, fentanyl has been defined more potent than heroin (∼50x) and morphine (∼100–300) for treating advanced cancer pain and surgery pain (1). However, same as in the case of heroine, high hydrophobicity of fentanyl opens a way for elicit use of this painkiller.

Drug abuse affects socioeconomic development globally, wailing for technologies to unmask advanced analytical solutions for decoding its biological complexity in humans (2, 3). Fentanyl and its analogs ignite an alarming social and health care crisis in form of addictive overdose followed by deaths world-wide in all ages, race, and sex (4). The hazardous substances data bank has described dose higher than 3 ng mL^−1^ of fentanyl as central nervous system (CNS) depressant, and the 10–20 ng mL^−1^ dosage responsible for anesthesia and profound respiratory depression in humans (5).

Fentanyl has a significant neurodegenerative impact on the brain, which is attributed to its high lipophilicity, enabling it passivating the brain–blood barrier (BBB) with ease (6, 7, 8). Once fentanyl crosses the BBB, it encounters the immune cells of the brain, namely, the microglial (HThμ) cells and astrocytes (NHA). Upon foreign moiety invasion, microglial cells are known for their signaling and phenotypic transformation, whereas astrocytes are more lipophilic, and both cells participate actively in cell debris cleaning mechanisms (9, 10, 11). Therefore, fentanyl accumulation in lipid droplets (LDs) in these cell lines likely triggers pathways for cellular death and therefore needs to be examined for molecular variations in response to fentanyl overdose.

Considering an extensive use of opioids as potent analgesics drugs as well as widespread opioids abuse, a comprehensive analysis of drug–cell interactions is of the outmost importance. However, conventional bioassays are not sufficiently developed for this research. A dye-based bioassay facilitates exploration of fentanyl overdose but identifying molecular-level changes in live single cell remains a challenge (12). Although colorimetric, fluorescence, electrochemical, mass-spectroscopy, and NMR-based techniques have been helpful for fentanyl’s detection, they do not support long-term monitoring of the drug in cells and its impact on biochemical pathways.

In our study, we have investigated the impact of fentanyl on the molecular composition of LDs in live human microglial and astrocyte cells—treated with a concentration of fentanyl that is reflective of a fentanyl overdose. Raman optical biosensing technique has been recently combined with molecular spectral unmixing software and bioinformatics tools giving rise to an optical Omics discipline coined Ramanomics (13, 14, 15, 16, 17, 18, 19). Ramanomics emerges as an excellent tool in identifying single organelle-based analysis of drug impact at subcellular levels which provides both qualitative and quantitative analysis of biochemical processes in various domains (20, 21). Our Ramanomics findings identify fentanyl induced changes in various chemical components (degree of lipid saturation, protein configuration, sphingomyelin and phosphocholine) and biological indicators such as lipid peroxidation. This unique single-cell Ramanomics approach provides a comprehensive view of changes in lipid metabolites in response to fentanyl that will help comprehend—not only the uptake of high concentration of fentanyl in the lipid membrane but also highlight the permeation process of fentanyl via its lipophilic binding affinity, which may explain its high potency and its neurotoxic potential.

## Materials and Methods

### Cells growth and fentanyl exposure

Fentanyl (Supelco, 1 mg/1 ml methanol) was procured from Ceriliant corporation Ltd. Basal cell culture media was purchased from ATCC Ltd. Normal human astrocytes (NHA) and human microglia cells (HMC3) were obtained from Cell application INC (882A-05a) and ATCC (cat. no. ATCC CRL-3304), respectively. The cells were cultured in luminescence-free 35 mm glass-bottom dishes (Fisher Scientific Co.). The culture medium used was Eagle’s Minimum Essential Medium (EMEM) (cat. no. ATCC 30–2003) supplemented with 5% foetal bovine serum (FBS), 100 units/ml penicillin, and 100 μg/ml streptomycin, and the cells were grown to 70% confluence at 37°C in a humidified atmosphere containing 5% CO_2_. The LDs were well distinguished under 100X magnification and subjected to laser irradiation respectively. A comparative analysis for overdose of fentanyl in cell lines was conducted using three (10, 100 and 1,000 ng mL^−1^) consecutive concentrations of standard fentanyl solutions.

### Cell staining BODIPY dye

Both the cell lines were cultured in 20 mm glass bottom cell culture dish (NEST ltd). Then, fentanyl-treated (30 min) cells were washed with PBS twice, followed by the addition of EMEM. Further, cells were stained by the BODIPY 581/591 C11 dye (5 μM) and then washed twice with PBS to be visualized under fluorescence microscope. Cells were subsequently washed and imaged in the green fluorescence channel, the fluorescent intensity was quantitated by using the ImageJ software (National Institutes of Health).

### Instrumentation

The spectra were measured on a DXR2 Raman microscopy setup (Thermo Fisher Scientific), equipped with a laser source unit emitting ∼60 mW at 633 nm (ROUSB-633-PLR-70-1, Ondax), a 50-μm pinhole to shape the laser beam to a 0.7 × 0.7 × 1.5 μm^3^ FWHM, and a Plan N 100× Olympus objective lens (NA = 1.25). In addition, the Raman microscope was equipped with a fluorescence lamp (X-Cite 120 PC, Photonic Solutions) to view live cells. Fluorescence imaging was done using the EVOS® FL Cell Imaging System (Life Technologies).

### Ramanomics assay

At first, live cells were obtained in an optically transparent Dulbecco’s Modified Eagle’s Medium (DMEM) (Thermo Fisher Scientific) and mounted onto the optical stage. To ensure a high-quality signal/noise ratio, the spectra accumulation parameter was set to 6 × 20 s with no measurable phototoxicity after irradiation dose. During the experiments, the cells were maintained under physiological conditions at 37°C. Spatial precision (XYZ position) of the LDs before and after each measurement was verified to attain absolute data in Raman spectra acquisition. Quantitative analysis of cellular spectra was performed using our BCAbox software (ACIS LLC). Detailed description of the method, output (BCA) parameters and the calibration of Raman band intensities on the concentrations of biomolecules in the sample were described in our previous publications (22, 23, 24, 25, 26).

## Results

**Fig. 1:**
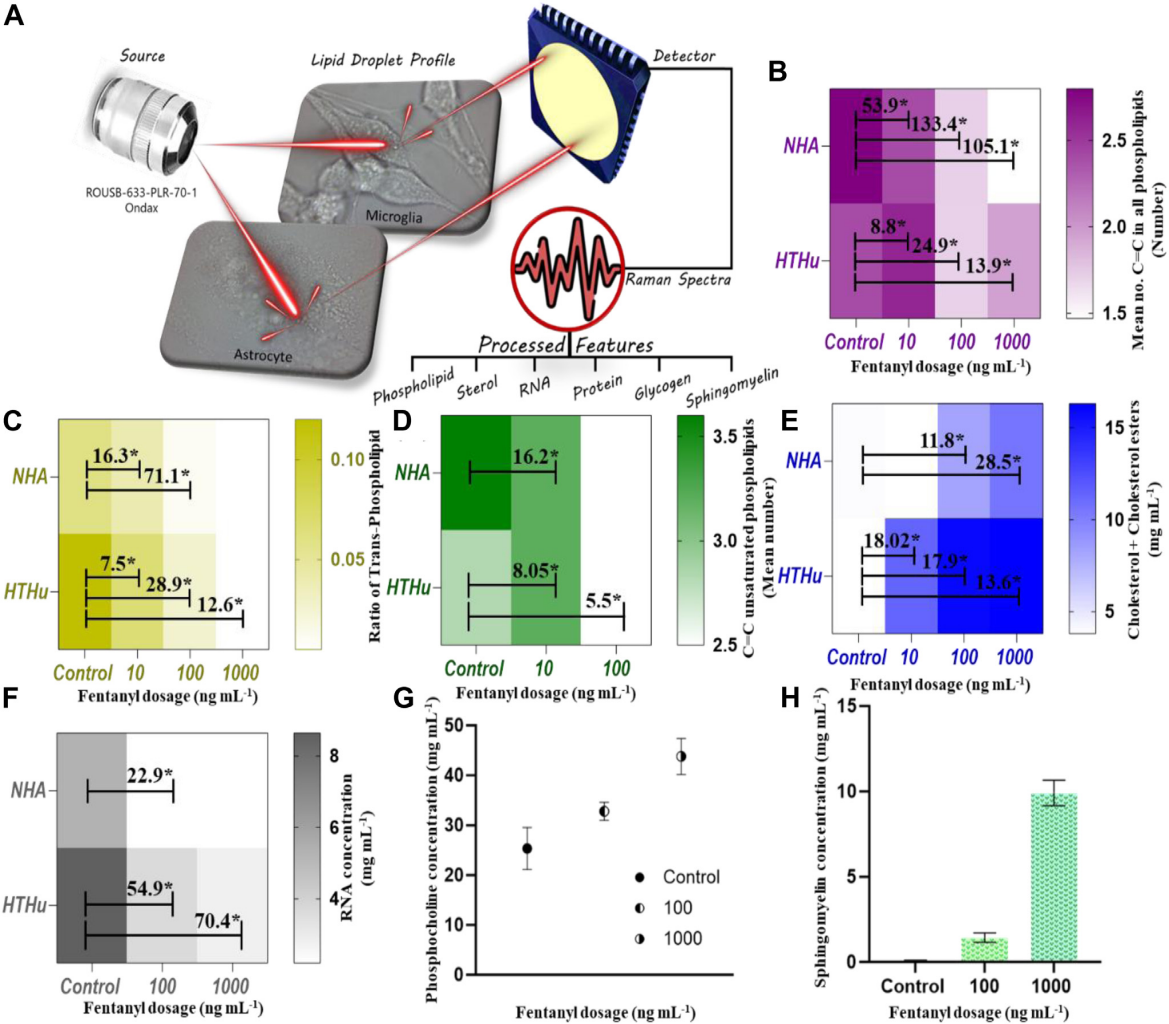
Schematic illustration of single cell lipid profiling after fentanyl overdose. A: ANOVA results for phospholipids features (B) mean number of C=C bonds, (C) ratio of trans-phospholipids, (D) mean number of C=C in unsaturated phospholipids; for sterols features (E) cholesterol and cholesterol esters and for (F) RNA content. Comparative analysis of change in (G) phosphocholine concentration and (H) sphingomyelin in human astrocytes with increase in fentanyl overdose.

Figure 1A illustrates the stepwise study conducted towards quantification of chemical features (phospholipids, cholesterol, glycogen, sphingomyelin, RNA, and LDs let surface proteins) using one-way ANOVA to BCA parameters. At first, the correlation between the dosage exposure time and spectral analysis was defined by varying the time duration of fentanyl susceptivity in microglial cells. Ten min, 30-min fentanyl exposure in both cell lines showed up distinct changes in pre-processed Raman signals (supplemental Fig. S1). The changes were observed at 699, 716, 764, 887, 926, 972, 1,265, 1,299, 1,593, 1,658, and 1746 cm^−1^ peaks with different overdose (10, 100, 1,000 ng mL^−1^) of drug treatment in the residual plot (supplemental Fig. S1B).

The comparative preprocessed Raman spectral data indicate significant changes in molecular bond specific Raman bands for each cell (supplemental Fig. S2). Under the region of phospholipids, the mean number of C=C in all phospholipids was found to drastically decrease in both human microglia and astrocytes (Fig. 1B). Saturation of phospholipids was ∼1.6 times higher in astrocytes in comparison to microglia, which can be attributed to more saturated lipid formation and oxidation of unsaturated lipids in human astrocytes in comparison to microglia under neurodegenerative stress (27). The human microglial cells showed a linear distinct difference in the ratio of trans-phospholipids with a variance of 12 folds with 1,000 ng mL^−1^. Trans phospholipids have linear fatty acid chains compared to their cis counterparts, which makes the membrane more rigid and less fluid, and this reduced fluidity can affect several membrane protein functions, membrane permeability, signal transduction, and membrane fusion processes such as vesicle formation, endocytosis, and exocytosis that rely on membrane flexibility. Alterations in the ratios of trans phospholipids can hinder these processes, affecting how cells interact with their environment. Further, Figure 1C showing the mean number of C=C bonds in unsaturated region of phospholipids confirms an increase of phospholipid saturation after exposure to overdose of fentanyl in both cell lines. Cholesterol and cholesterol ester, an important constituent of LDs, were found to elevate in concentration with the fentanyl exposure content. In the case of microglia, the increase was distinct at the 10-ng mL^−1^ exposure, with a fold of 2.5 when compared to astrocytes. The accumulation of cholesterol esters was a vital observation in our findings as neuroscience associated to its accretion has been found to trigger cell death of microglial cells (28).

Glycogen has a heterogeneous pattern of distribution within the brain (29) and is observed in diverse cell types within the central nervous system (CNS), including neurons, astrocytes, and microglia (30, 31). The majority of brain glycogen is in astrocytes, which is utilized as an energy reserve to support neurons when glucose availability is limited. Glycogen is a critical component of cerebral metabolism. We observed that in brain astrocytes, there is significant accumulation of glycogen which contributes to lipids metabolism. Glycogen contributes to lipid metabolism by serving as a source of glucose which can be converted into fatty acids, enabling storage of carbohydrates via novo lipogenesis. We found a significant increase in glycogen, at 100 and 1,000 ng mL^−1^ fentanyl exposure, which suggests that when cells undergo stress, excess glucose produced gets accumulated as glycogen in LDs in astrocytes (supplemental Fig. S3). Glycogen accumulation-based LD biogenesis predominantly occurs in liver and has been observed in adipocytes and pre-adipocytes (32, 33); however, brain glycogen plays an important role in learning and memory, signaling events, neurotransmitter metabolism, and protein glycosylation (34). Glycogen metabolism directly supports neuronal and astrocytic functions and mainly supports brain energy metabolism. Glycogen is also found in microglia, and in our study, microglia shows a decrease in glycogen from 7.02 to 0.18 mg/ml as compared to an increase from 1.54 to 2.35 mg/ml in astrocytes, which may be due to its higher involvement in cell signaling and phagocytic pathways to address the drug lethal impact (35). The RNA content was found to decrease across LDs after the exposure of fentanyl, which can be attributed to decreased transcription, increased RNA degradation due to cellular stress, potentially leading to impaired protein synthesis and disrupted cellular function under conditions of oxidative stress (Fig. 1F). In addition to this, the amount of surface proteins (class I and II) in LDs for astrocytes was found to increase sufficing for more LD formation, whereas its decrease in microglia can be inferred as deterioration in LDs when exposed to fentanyl overdose (supplemental Fig. S4).

Distinct molecular variations were observed in microglia and astrocytes in response to fentanyl. All fentanyl treatment resulted in an increase in the concentration of phosphocholine in microglial cells (Fig. 1G). Human C-reactive protein is a cyclic oligomer, which binds to phosphocholine in a Ca^2+^-dependent manner and plays an important role in protection against infection, clearance of damaged tissue, prevention of autoimmunity as well as in regulation of the inflammatory response (36). An increase in the concentration of phosphocholine for fentanyl- treated microglial cells can be correlated with the inflammatory immune response property of microglial cells, triggered after drug exposure. But in the case of astrocytes, it was quite evident that no such specific change in phosphocholine was observed, confirming its limited role in inflammatory immune response. In astrocytes, there was an increase in the sphingomyelin concentration on exposure to increased dose of fentanyl (Fig. 1H). Sphingomyelin has been shown to influence LD formation, and in accordance with our findings, it was observed that astrocyte LDs increased drastically in comparison to untreated cells (37, 38, 39, 40, 41). This observation was also evident in the bright field images during the Raman study (supplemental Fig. S5). Such an increase in sphingomyelin can be attributed to increased synthesis of more LDs by the cell.

**Fig. 2:**
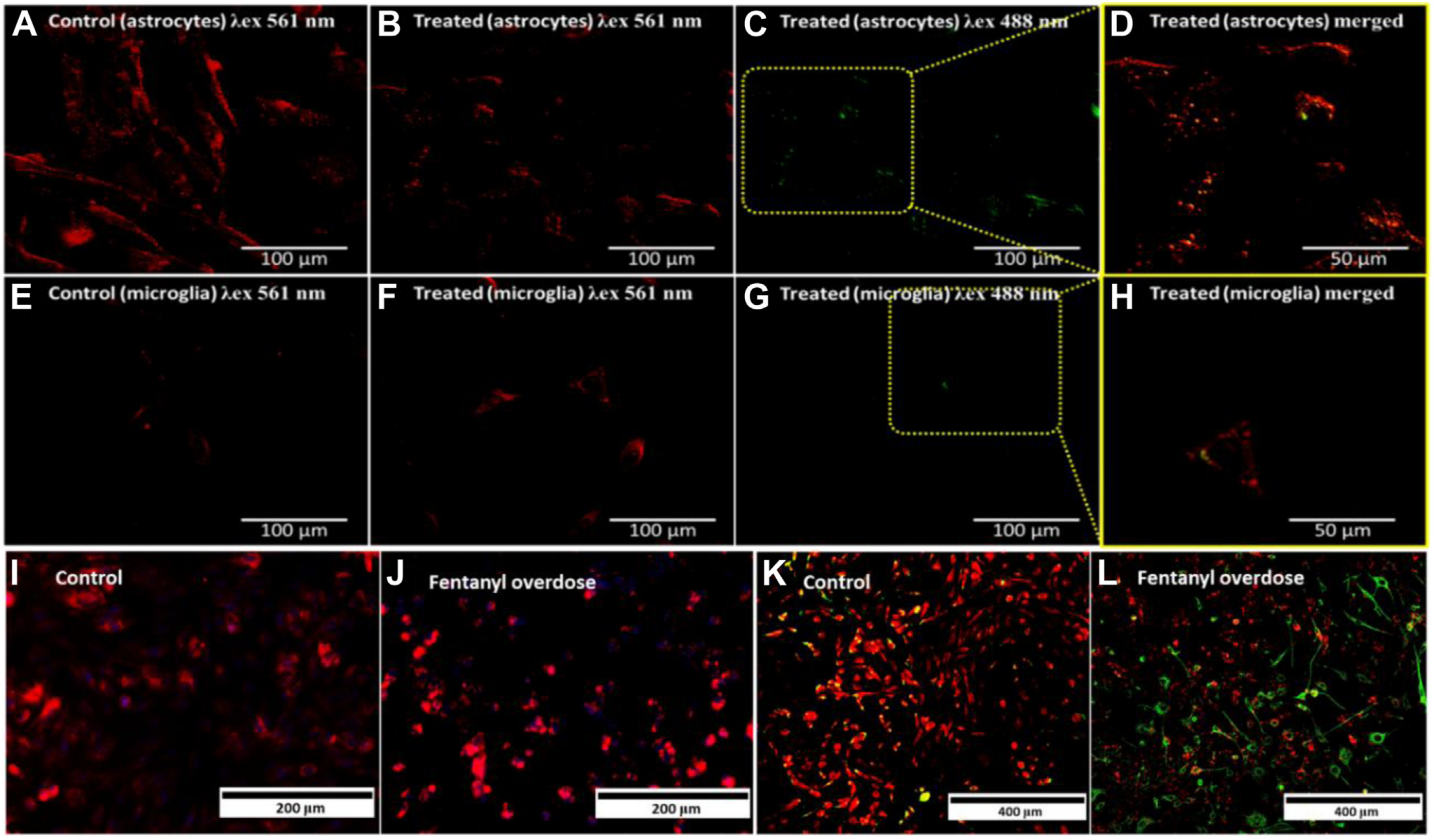
Fluorescence imaging for fentanyl (100 ng mL^−1^)-induced lipid peroxidation in human astrocytes and microglia. Lipid peroxidation (BODIPY 581/591 C11 dye) identification in astrocytes, control (A) λ_ex_ = 561 nm: λ_em_ = 600 nm (B) λ_ex_ = 488 nm: λ_em_ = 510 nm and fentanyl treated (C) λ_ex_ = 561 nm: λ_em_ = 600 nm, (D) λ_ex_ = 488 nm: λ_em_ = 510 nm and in microglia, control (E) λ_ex_ = 561 nm: λ_em_ = 600 nm, (F) λ_ex_ = 488 nm: λ_em_ = 510 nm and 100 ng mL^−1^ fentanyl treated (G) λ_ex_ = 561 nm: λ_em_ = 600 nm, (H) λ_ex_ = 488 nm: λ_em_ = 510 nm. Astrocytes calcium ion regulation in (I) control and (J) fentanyl overdosed cells. IBA1 stained co-culture (astrocytes and microglia) in (K) control and (L) fentanyl overdosed cells for changes in calcium ion regulation.

Lipid peroxidation refers to the oxidative degradation of cellular lipids by free radicals from reactive oxygen species, which carry the ability to break the C=C bonds in phospholipids and polyunsaturated fatty acids, resulting in initiating cell death pathways (42). LDs in fentanyl-treated microglial cells and astrocytes were stained with BODIPY dye. Cellular Staining using the BODIPY Red dye enabled quantitation of Lipid Peroxidation in fentanyl treated microglia and astrocytes (Fig. 2). With the increase in fentanyl dosage, the dye fluorescence intensity in the green region under 488 nm excitation increased, affirming the peroxidation of unsaturated fatty acid chains (Figs. 2B–D in astrocytes, 2F–H in microglia). We infer that reactive oxidative stress that is generated by fentanyl treatment is attributed to the enhanced lipid peroxidation process in both microglia and astrocytes (43). The increased lipid peroxidation as indicated by BODIPY staining was affirmatory and corroborated with the information derived from the Ramanomics analysis fentanyl-induced changes in LDs.

Additionally, the crucial role of calcium ion regulation with respect to drug increase in both cell lines was investigated to understand the mechanism at ion level. Human astrocytes exposed to fentanyl overdose showed an increase in Ca^2+^ regulation (Fig. 2I, J). The distinct IBA1 marker (microglia specific) was used to differentiate and derive insights onto calcium regulation in co-culture (astrocytes and microglia) treated with fentanyl (100 ng mL-1). It exhibited a decrease in Ca^2+^ regulation in microglia, whereas in astrocytes, it was found to increase with an increase in drug dose. Upon elevated calcium concentration, augmentation in oxidative stress by glutamatergic N-methyl-D-aspartate receptor activation leads to astrocyte death. In the case of microglia, the downregulation of calcium ion can be attributed to blocking of the calcium ion channel.

**Fig. 3:**
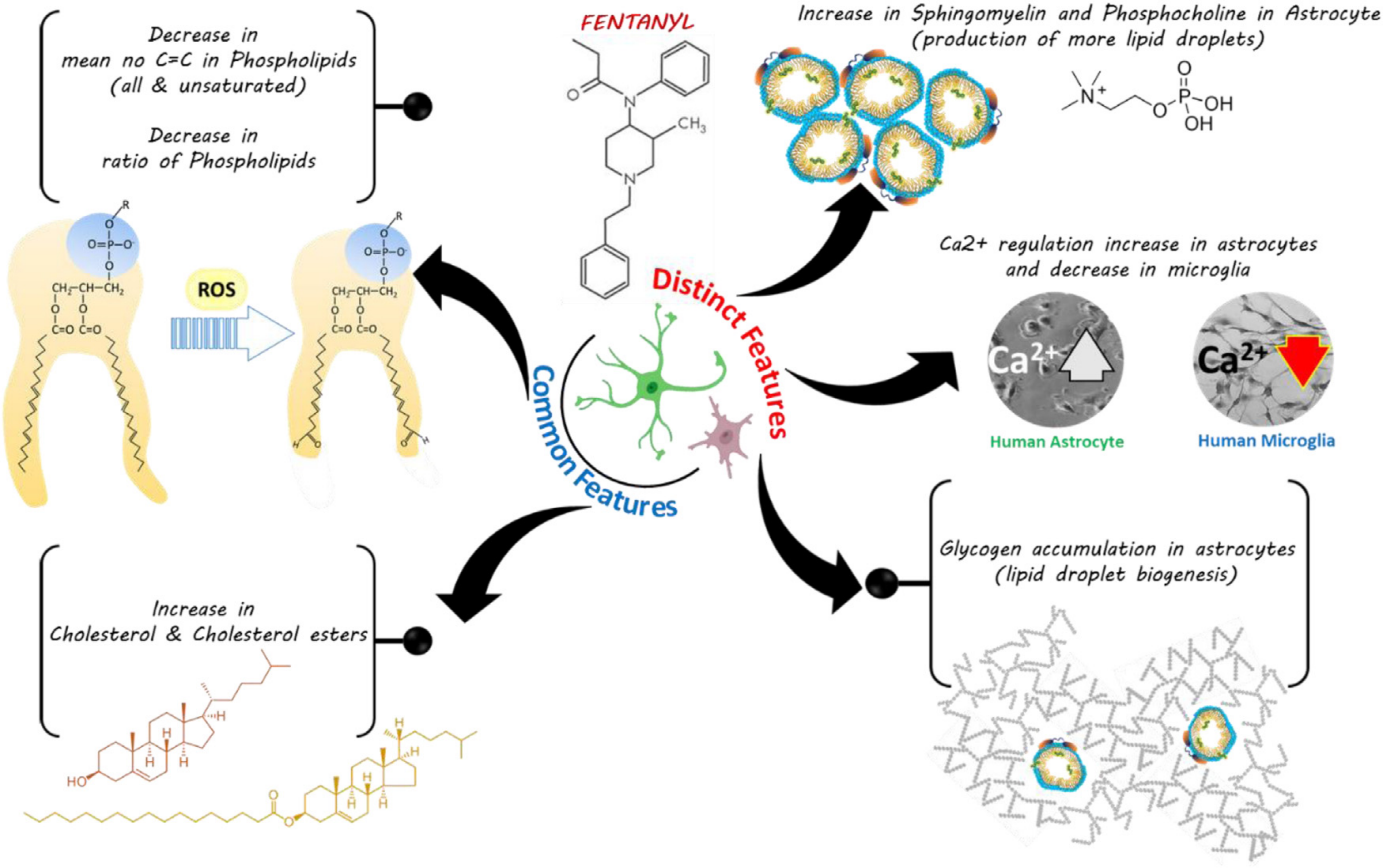
Proposed interpretation of fentanyl induced toxicity in astrocytes and microglial cells.

Figure 3 provides an insight into common and distinct features derived from the Ramanomics analysis of fentanyl-treated human astrocytes and microglia 30 min post treatment.

## Discussion

Based on our Ramanomics analysis, fentanyl-induced decrease in the number of C=C bonds in phospholipids indicates a loss of unsaturation and a decrease in carbon atoms in astrocytes and microglia. We speculate that this fentanyl-induced change in the degree of fatty acid unsaturation in membrane phospholipids may affect many crucial membrane-associated functions, such as modulation of ion channels like Ca^2+^ channel shown in Figure 2 and processes such as endo- and exocytosis that are key to membrane barrier functions. Additionally, an alteration in the degree of fatty acid unsaturation in cell membranes is also implicated in a variety of neurological diseases. Several reports suggest that fatty acids in membrane phospholipids of mammalian cells exhibit considerable structural diversity, including varying chain lengths and degrees of unsaturation which modulate membrane-associated functions. Thus, the degree of fatty acid unsaturation in membrane phospholipids may determine the biophysical properties of the membrane (44, 45, 46, 47, 48).

Fentanyl treatment leads to accumulation of g in astrocytes and microglia. Brain glycogen plays a key role in learning and memory, signaling events, neurotransmitter metabolism, and protein glycosylation (34). An important constituent of brain glycogen is Glucosamine which contributes to protein glycosylation in the brain. Glycosylation defects are a hallmark of many neurological disorders, and thus fentanyl-induced changes in glycogen metabolism modulates neuronal and astrocytic functions via altering brain energy metabolism via when glucose availability is reduced in conditions such as neurological stress, dementia or traumatic brain injury.

Fentanyl increased phosphocholine levels in astrocytes and increased phosphocholine levels in the brain are associated with brain injury and neurodegenerative diseases indicating potential cell damage or abnormal cell metabolism. Phosphocholine is a breakdown product of phosphatidylcholine, which plays a crucial role in regulating lipid and lipoprotein levels in the CNS (49). Choline-based phospholipids are involved in maintaining the structural integrity of the neuronal/glial cell membranes and are essential components of various biochemical pathways, such as cholinergic neuronal transmission in the brain (50).

Fentanyl-induced increase in sphingomyelin is associated with neurodegenerative disease progression, and increased sphingomyelin can result in elevated ceramide levels which is implicated in neuronal damage (51).

Fentanyl induces an increase in cholesterol and cholesteryl esters in astrocytes and microglia, and this accumulation of these lipids in these cells can contribute to brain damage, cognitive decline, and progression of neurodegenerative diseases. Although cholesterol and cholesteryl esters are essential to healthy brain function, excessive cholesterol, particularly in the form of cholesteryl esters, can be detrimental to the brain (52). Furthermore, accumulation of cholesterol and cholesteryl esters in astrocytes and microglia can also impair clearance mechanisms within the brain, leading to an inflammatory response. Fentanyl can affect cytoplasmic LDs by altering the composition and abundance of lipid species within neural cells. High dosage of the drug is associated with increased levels of ceramide and hexosylceramide, which suggest alterations in lipid metabolism that result in increased cellular stress and apoptosis, resulting in neurotoxicity and neurocognitive impairments due to fentanyl abuse. Fentanyl-induced changes in LDs can exacerbate neuroinflammatory responses, leading to further neuronal damage (53). Furthermore, the activation of microglia and astrocytes, which are central to the inflammatory response in the brain, can result in the release of pro-inflammatory cytokines and reactive oxygen species resulting in chronic inflammation that can disrupt neuronal function and contribute to the development and progression of neurodegenerative diseases (54, 55, 56).

As outlined previously, distinct and general features associated with the fate of phospholipids were loss of unsaturation and a decrease in carbon atoms (57, 58). Increase in sterols and ester derivatives are discussed in the context of both CNS cell types evaluated in this study. LDs are dynamic organelles that play a crucial role in lipid metabolism, energy storage, and cellular signaling influencing key pathways such as the PI3K/Akt/mTOR and Wnt/β-catenin pathways, which are associated with neurodegeneration. Alterations in LDs can have significant implications for both lipid metabolism and neurodegenerative diseases. Dysregulation of lipid metabolism, including the synthesis, transport, and utilization of lipids, has been implicated in the pathogenesis of several neurodegenerative diseases (59). LDs are involved in the storage and hydrolysis of neutral lipids, such as triacylglycerol (TAG) and cholesterol esters (CE). Alterations in LDs can influence the risk of developing metabolic diseases, such as obesity, diabetes, and non-alcoholic fatty liver disease. LD-associated proteins, such as perilipins and diacylglycerol acyltransferase (DGAT), play a critical role in the biogenesis, maturation, and catabolism of LDs. Dysregulation of these processes can lead to metabolic imbalances and contribute to the development of metabolic disorders (60). LDs are not only passive lipid storage compartments but also play an important role in the initiation and progression of diverse pathophysiological processes, such as stress, neuroinflammation, and energy metabolism, in the CNS under conditions of neurodegeneration, neuroinflammation and aging. Microglia and astrocytes containing LD exhibit distinct functional phenotypes, which can either confer protection against neurological diseases or exacerbate CNS disease progression. (61, 62). LDs are involved in various cellular processes, including oxidative stress response, lipid peroxidation, and the regulation of reactive oxygen species (ROS) levels, and these processes can either facilitate cell survival or lead to cell apoptosis. The found fentanyl-induced changes in the levels of phosphocholine, sphingomyelin cholesterol esters, and glycogen levels in microglia and astrocyte demonstrate the utility of Ramanomics analysis in differentiating the role of these cell in brain metabolism but can also have potential applicability in therapeutic targeting of neurological disorders.

## Conclusion

In summary, the various parameters analyzed by the Ramanomics approach helped derive novel information related to chemical constituents of LDs within an individual microglial or astrocytic cell treated with fentanyl at single-organelle level. Under the influence of fentanyl overdose, the degree of saturation (C=C) in all phospholipids, cholesterol with its esters and glycogen accumulation on LDs were common but quantitatively different in both cell types. Additionally, the concentrations of sphingomyelin, trans-phospholipids, and phosphocholines are increased post fentanyl exposure. Moreover, surface proteins (Class I and II) were also found to deteriorate on LD surface, supporting a collapse of LDs, whereas the RNA content was found to decrease with an increase in fentanyl dose. Distinct features such as elevation of phosphocholine and sphingomyelin concentration in individual microglia or astrocyte in response to fentanyl treatment demonstrate the utility of Ramanomics analysis as a non-invasive, real-time analysis of cellular/molecular changes in response to a drug stimulus. Information generated from this study will help guide therapeutics advances to treat fentanyl addiction and be of importance in future design of new fentanyl-based analgesic/anesthetic drugs used to treat chronic pain.

## Data availability

The data that support the findings of this study are available from the corresponding author upon reasonable request.

## Supplemental data

This article contains supplemental data.

## Conflict of interest

The authors declare that they have no conflicts of interest with the contents of this article.
