# Supplementary figures and images for: Fentanyl-induced transformations in composition of lipid droplets in central nervous system cells revealed by ramanomics
Source: J Lipid Res. 2025 May 19;66(7):100827. doi: 10.1016/j.jlr.2025.100827 (PMC12221884; doi:10.1016/j.jlr.2025.100827)

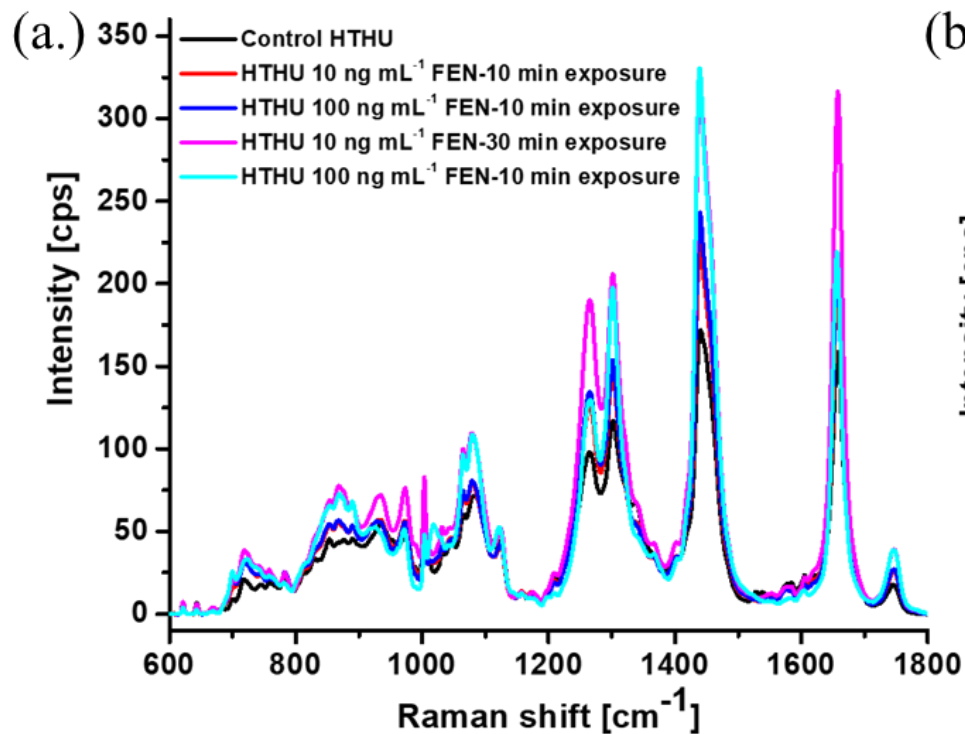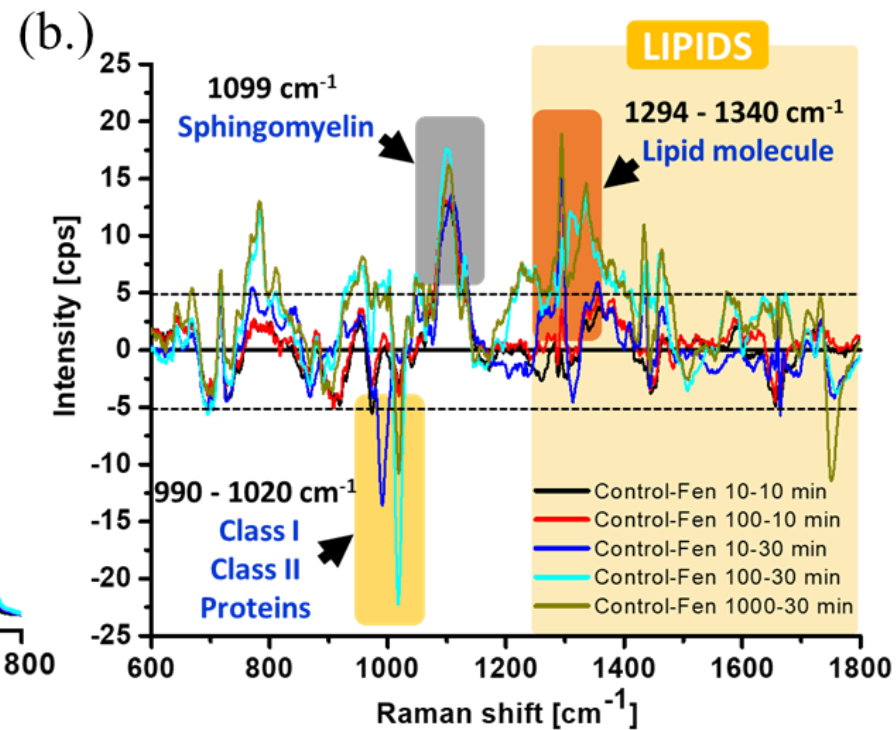

Supplement: Fig S1 [file mmc1.pdf]

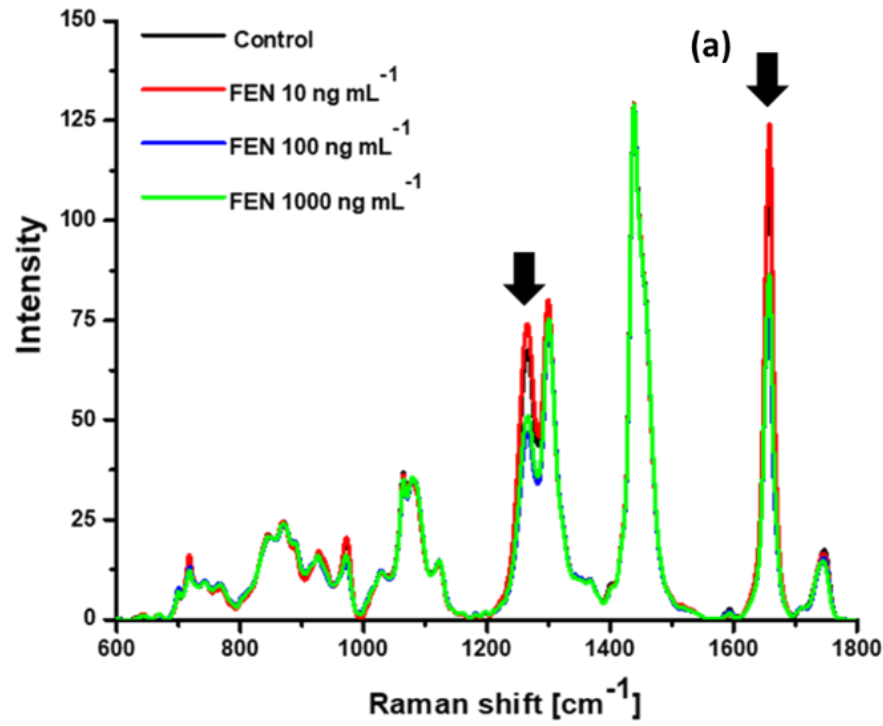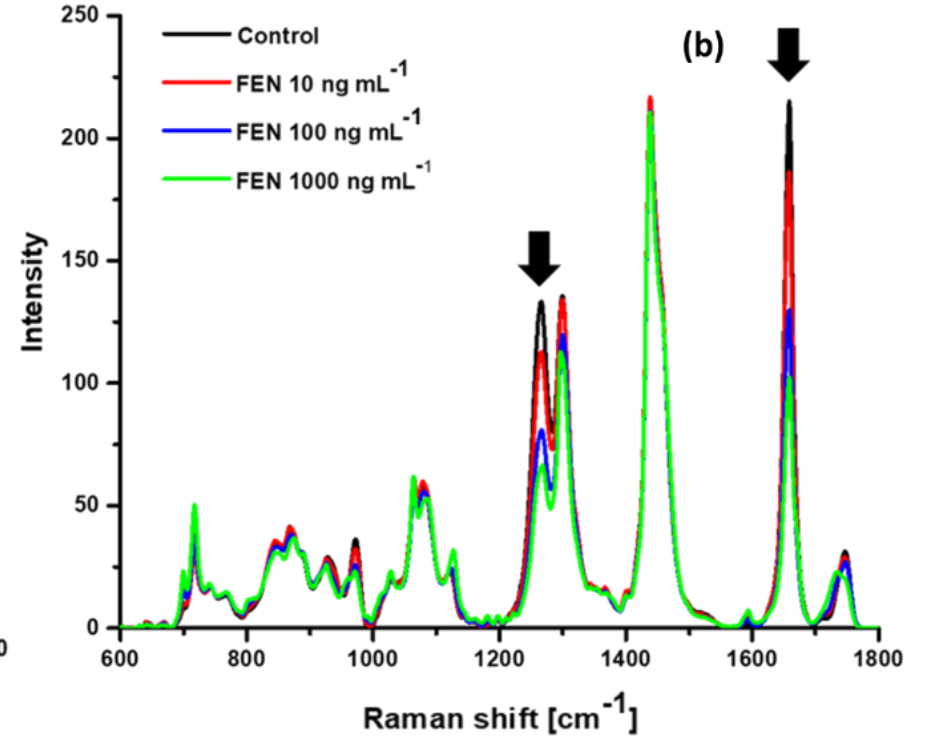

Supplement: Fig S2 [file mmc2.pdf]

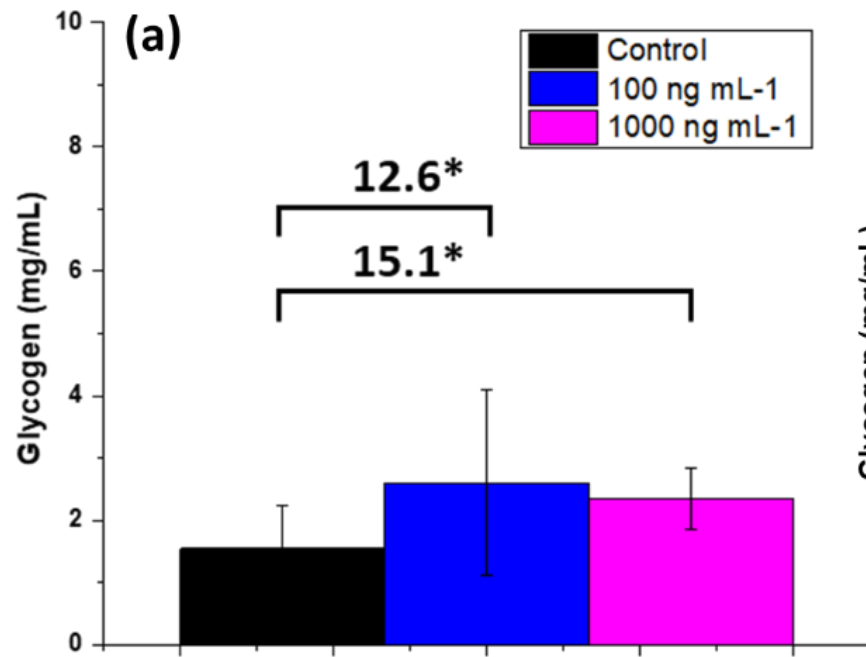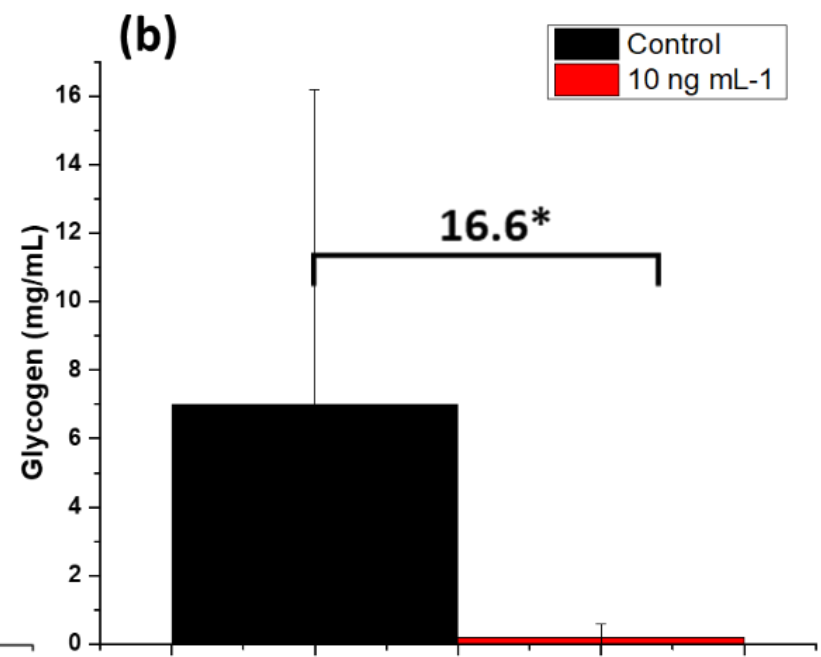

Supplement: Fig S3 [file mmc3.pdf]

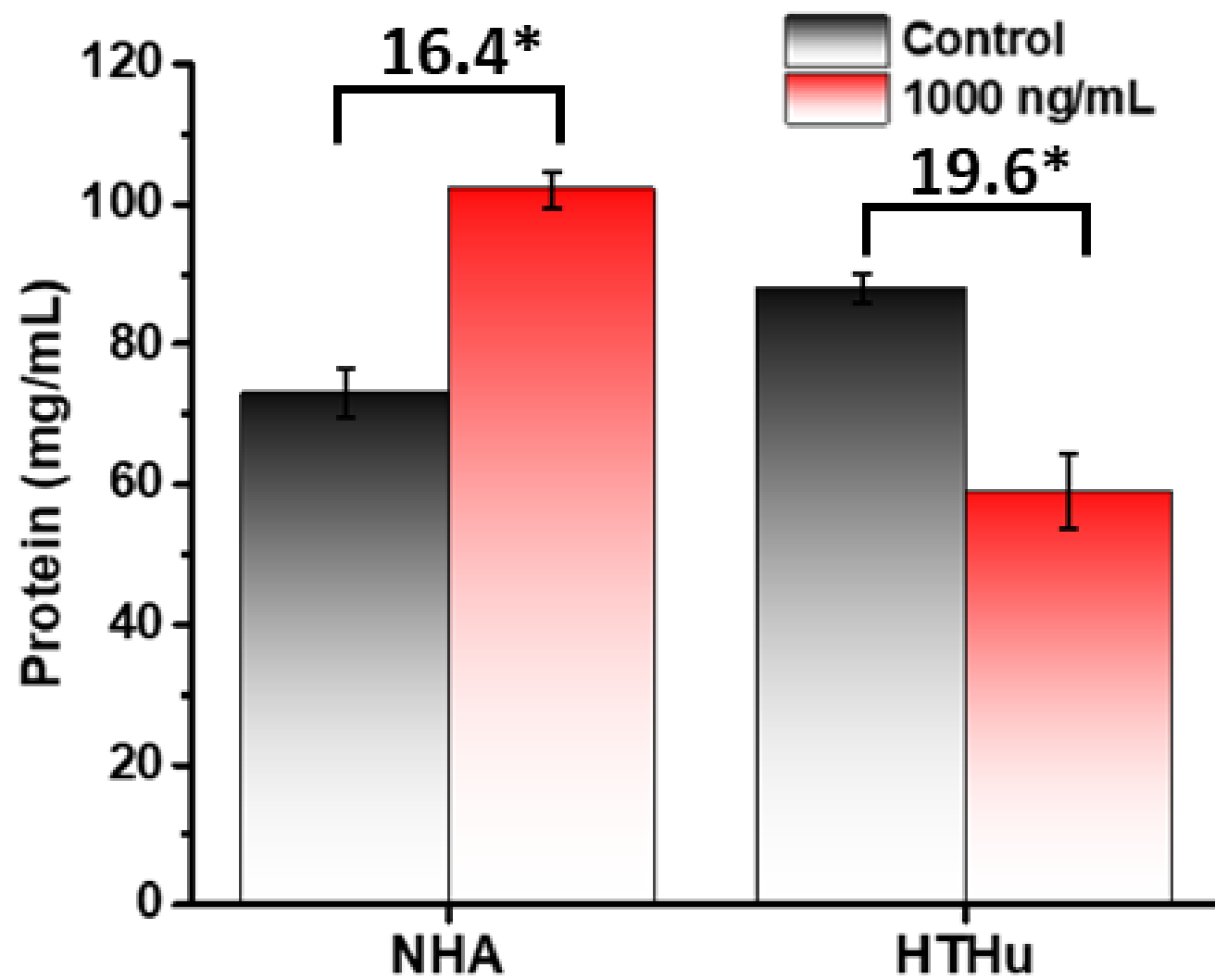

Supplement: Fig S4 [file mmc4.pdf]

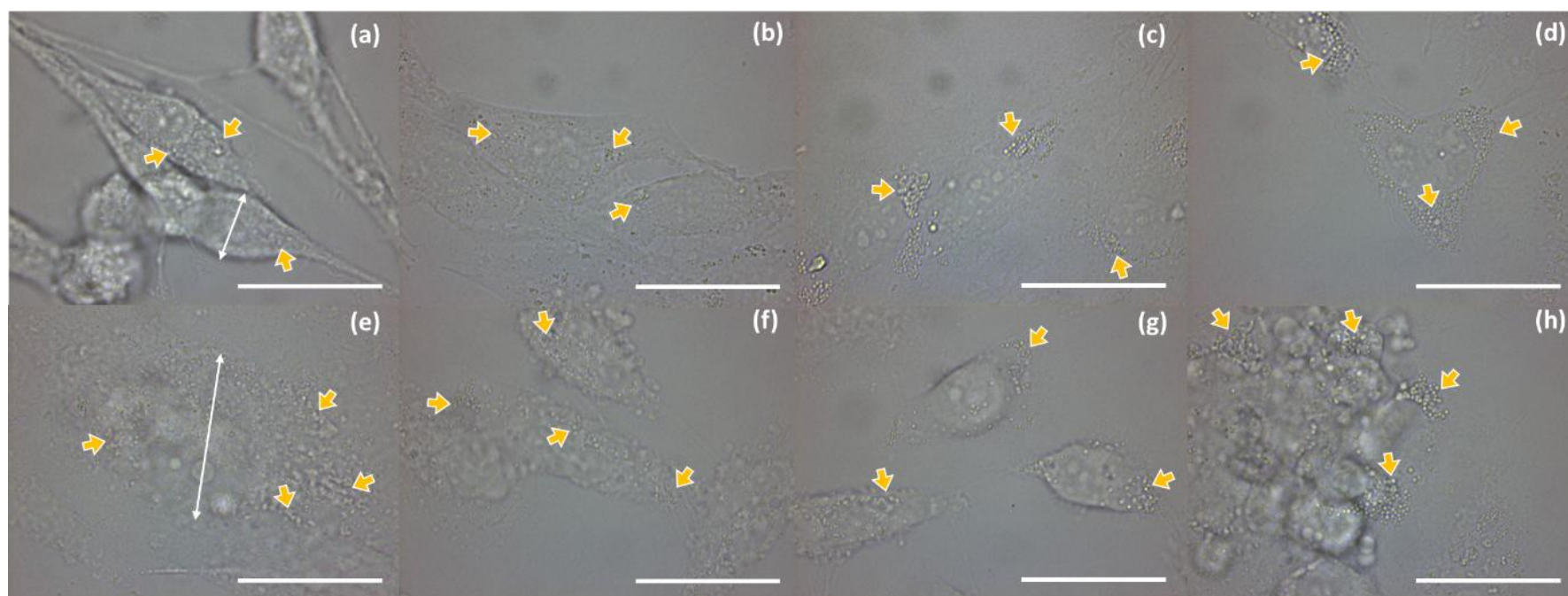

Supplement: Fig S5 [file mmc5.pdf]
