# Supplementary material for: Fentanyl-induced transformations in composition of lipid droplets in central nervous system cells revealed by ramanomics
Source: J Lipid Res. 2025 May 19;66(7):100827. doi: 10.1016/j.jlr.2025.100827 (PMC12221884; doi:10.1016/j.jlr.2025.100827)
Supplement: Supplementary Information [file mmc6.docx]

**Supplementary Information**

**Ramanomics of Lipid Droplets in Fentanyl Overdosed Central Nervous System Cells**

Rahul K. Das,^1^ Andrey N. Kuzmin,^1*^ Artem Pliss, ^1,2^ Supriya D. Mahajan,^3^ Shobha Shukla,^4^ Paras N. Prasad^1^*

^1^Institute for Lasers, Photonics and Biophotonics and Department of Chemistry, University at Buffalo, The State University of New York, Buffalo, New York 14260, United States

^2^ School of Pharmacy, D’Youville University, Buffalo, New York 14201, United States

^3^Department of Medicine, Division of Allergy, Immunology, and Rheumatology, State University of New York at Buffalo, Clinical Translational Research Center, Buffalo, New York 14203, United States

^4^Nanostructures Engineering and Modeling Laboratory, Department of Metallurgical Engineering and Materials Science, Indian Institute of Technology Bombay, Mumbai 400076, MH, India

*For correspondence: Andrey N. Kuzmin, [ankuzmin@buffalo.edu](mailto:ankuzmin@buffalo.edu); Paras N. Prasad, pnprasad@buffalo.edu

**
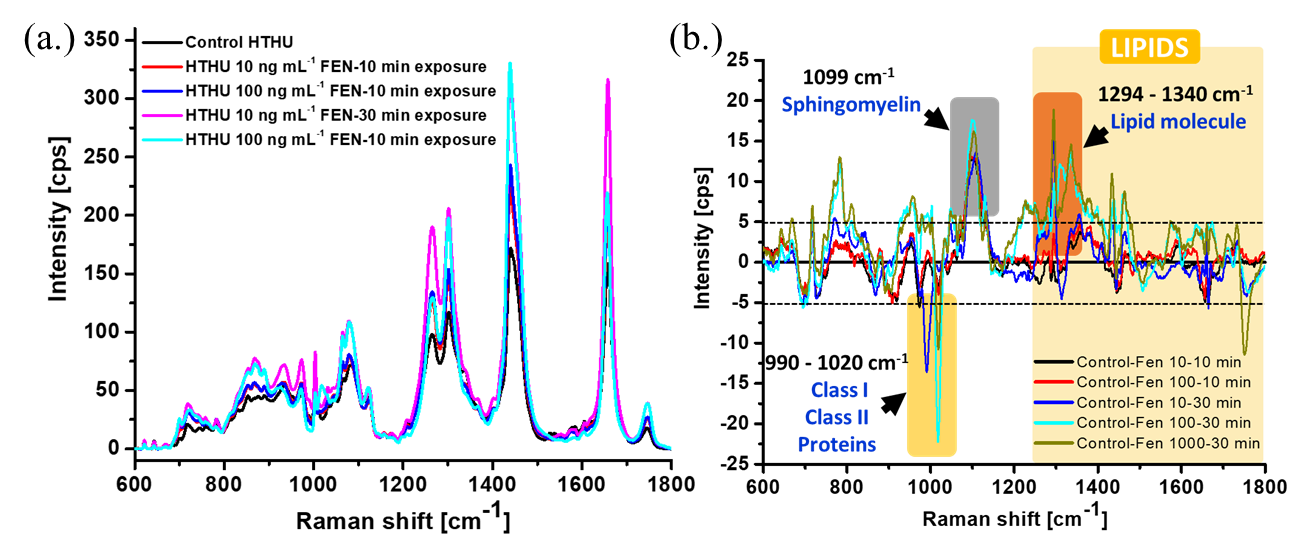
**

***Fig S1.*** *Comparative (a) Pre-processed and (b) residual Raman spectra for fentanyl overdosed microglia cells with drug exposure time of 10 and 30 minutes.*

**
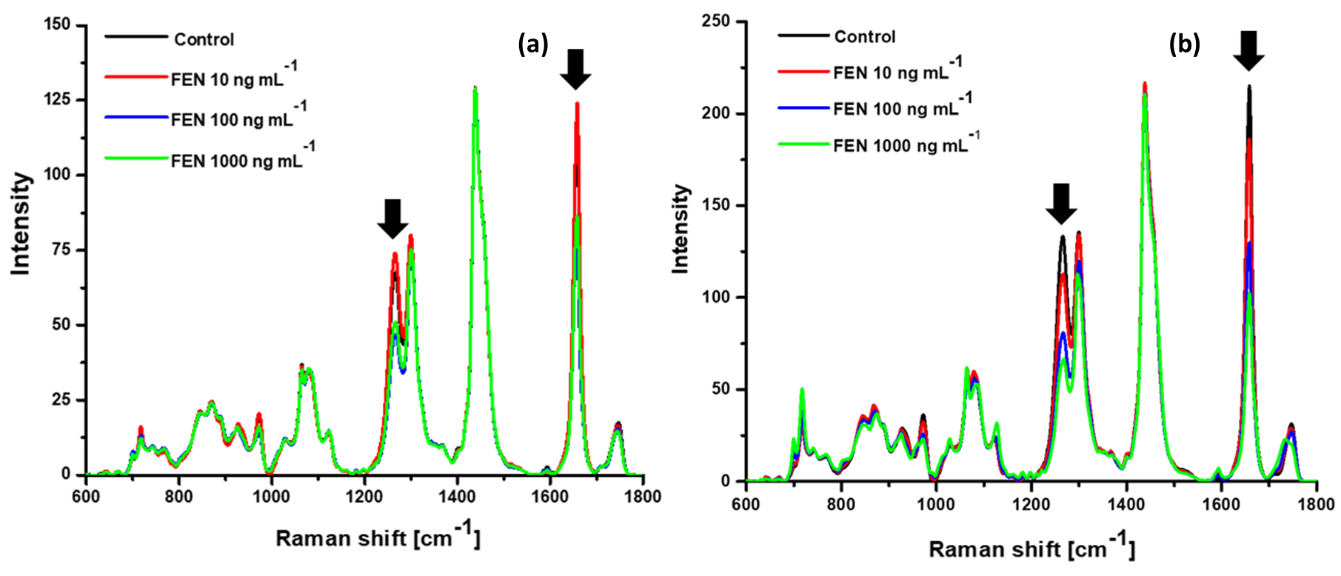
*Fig S2.*** *Raman spectra of lipids, extracted from LD Raman spectra by BCA, for human (a) microglial and (b) astrocytes treated with different overdose of fentanyl.*

*
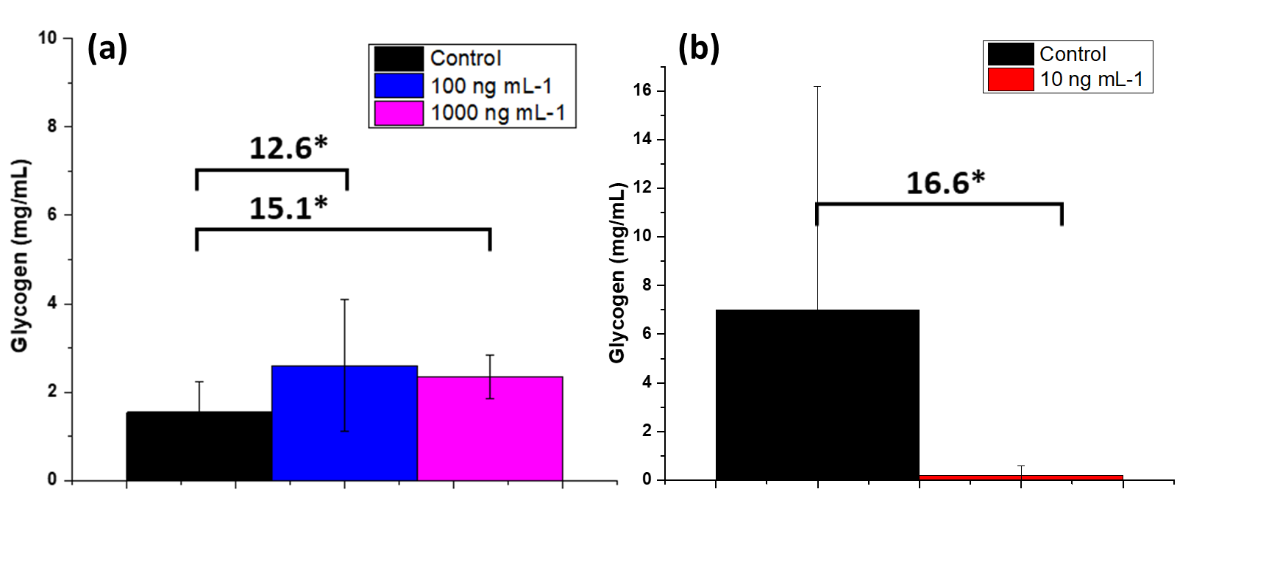
*

***Fig S3.*** *ANOVA results for change in glycogen content in astrocytes and microglia under different overdose of fentanyl.*

*
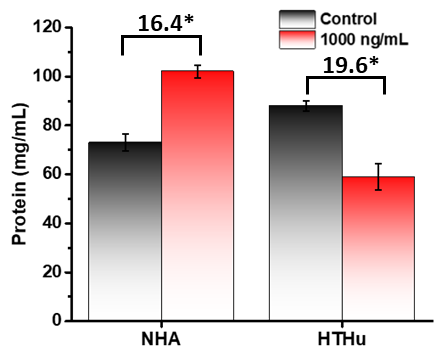
*

***Fig S4.*** *ANOVA results for the following BCA parameters protein content in astrocytes and microglia*

**
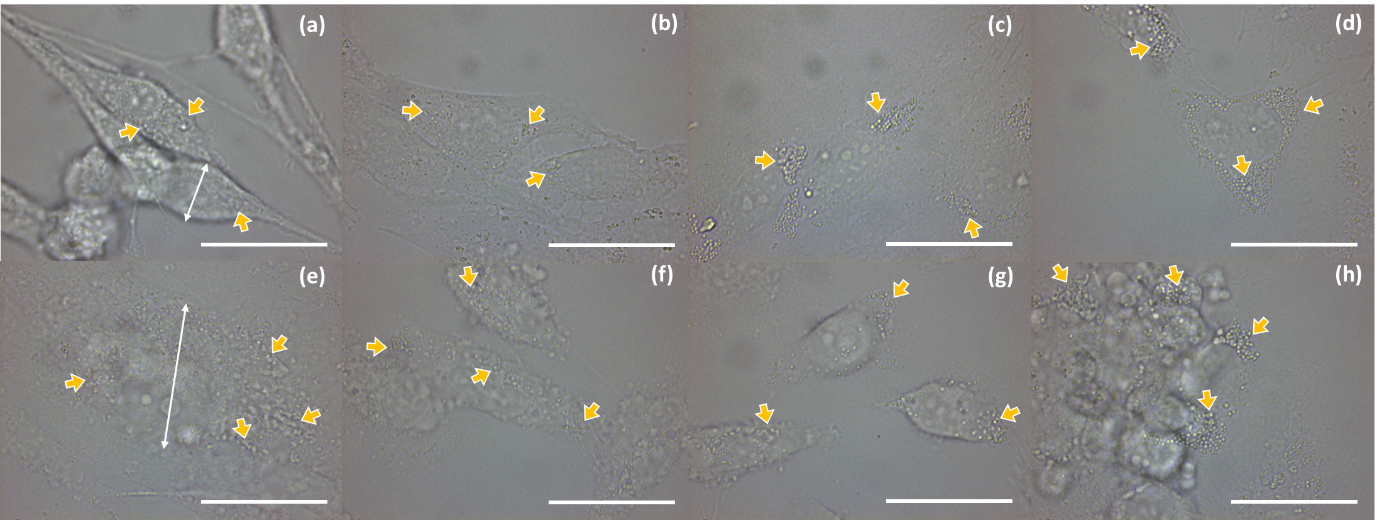
**

***Fig S5.*** *Bright field images (100 X) of microglia (a) control and treated with fentanyl (b) 10, (c) 100, (d) 1000 ng mL-1. Bright field images (100 X) of astrocytes (a) control and treated with fentanyl (b) 10, (c) 100, (d) 1000 ng mL-1 overdose. (marking shows LD distribution; scale 50 μm)*
